# Unraveling the rapid CO_2_ mineralization experiment using the Paraná flood basalts of South America

**DOI:** 10.1038/s41598-024-58729-w

**Published:** 2024-04-06

**Authors:** Alanielson Ferreira, Roberto Ventura Santos, Tarcísio Silva de Almeida, Maryene Alves Camargo, José André Filho, Caetano Rodrigues Miranda, Saulo de Tarso Alves dos Passos, Alvaro David Torrez Baptista, Colombo Celso Gaeta Tassinari, Valentina Alzate Rubio, Gabriel Godinho Capistrano

**Affiliations:** 1 Instituto de Geociências, Universidade de Brasília, Brasília, DF Brazil; 2 Instituto de Química, Universidade de Brasília, Brasília, DF Brazil; 3 Instituto de Física, Universidade de São Paulo, São Paulo, SP Brazil; 4 Research Centre for Greenhouse Gas Innovation, São Paulo, SP Brazil; 5 Instituto de Geociências, Universidade de São Paulo, São Paulo, SP Brazil; 6 Brasília, DF Brazil

**Keywords:** Carbon Capture and Storage (CCS), CO_2_ geostorage, Basaltic reservoirs, Paraná continental flood basalts, Experimental petrology, Geochemistry, Environmental chemistry, Mineralogy, Petrology

## Abstract

CO_2_ capture and storage in geological reservoirs have the potential to significantly mitigate the effects of anthropogenic gas emissions on global climate. Here, we report the results of the first laboratory experiments of CO_2_ injection in continental flood basalts of South America. The results show that the analyzed basalts have a mineral assemblage, texture and composition that efficiently allows a fast carbonate precipitation that starts 72 h after injection. Based on the availability of calcium, chemical monitoring indicates an estimated CO_2_ storage of ~ 75%. The carbonate precipitation led to the precipitation of aragonite (75.9%), dolomite (19.6%), and calcite (4.6%).

## Introduction

Mitigating CO_2_ emission levels is a crucial issue and has been linked to the life conditions of our times^1–4^. Carbon Capture and Storage (CCS) methods are one of the most promising technologies for reducing anthropogenic CO_2_ emissions^5–7^. Among the available strategies, storing carbon in soils and unstable geologic formations (i.e., exhausted hydrocarbon fields, saline aquifers) have critical difficulties related to the long-term stability of the reactions and physical states of CO_2_, unknown effects arising from geotechnical instabilities and constant risks of erosion and CO_2_ leakage^1,5,7–9^. In contrast, strategies based on basaltic formations have several advantages for carbon storage, including high reactivity of minerals with CO_2_, considerable storage potential due to the vast volume of subsurface basalt, and potential for rapid reaction kinetics and long-term storage of CO_2_^10–13^.

Permanent CO_2_ geostorage or mineralization converts carbon dioxide into inert crystals by precipitating carbonate in subsurface basaltic rocks^2,3,13^. Flood basalts are common igneous rocks on the surface of continents, usually related to Large Igneous Provinces^14^. The high content of CaO (6–12 wt.%) and MgO (4–10 wt.%) turn basaltic rocks more reactive when compared to siliciclastic sedimentary rocks, mainly in the presence of surface fluids as carbonic acid and even meteoric waters^1,12^. Available studies demonstrate that natural reactions involving basalts on the Earth’s surface account for 30–35% of natural CO_2_ sequestration from the atmosphere by weathering^15,16^. In light of this, major CCS projects currently use basaltic rocks, like Carbfix projects in Iceland^2–4^ and Wallula project in the United States^17^.

**Figure 1:**
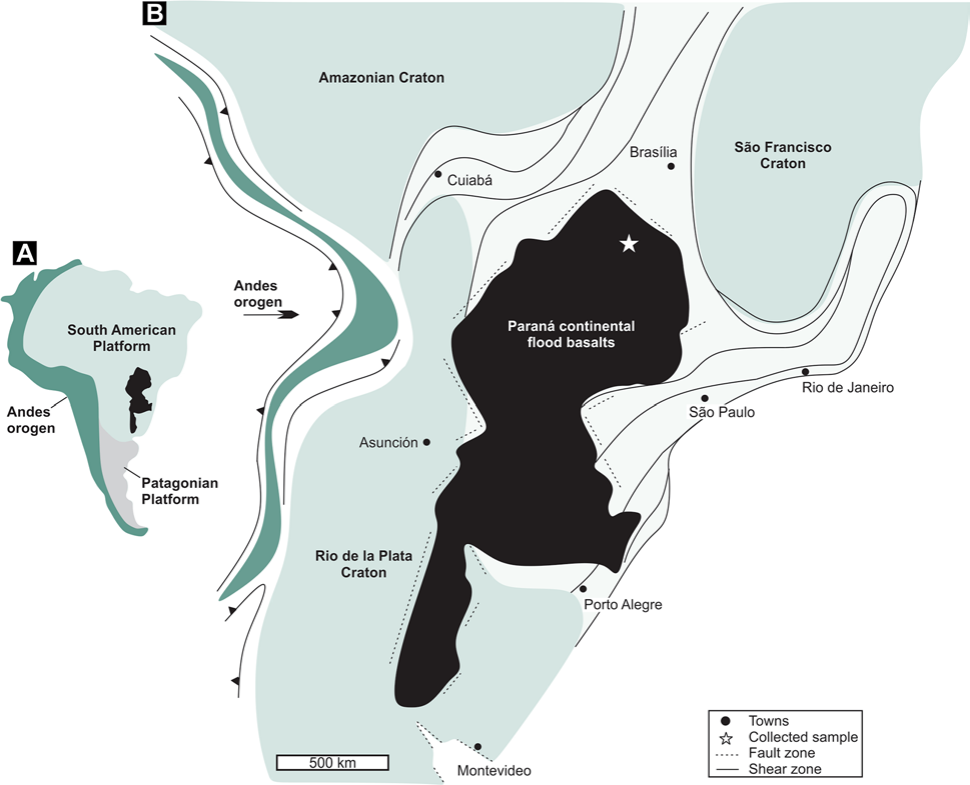
(**A**) Major geodynamic units of South America. (**B**) Geology map with the spatial distribution of the Paraná continental flood basalts in the southern portion of the South America platform^24^.

In South America, there are few CCS projects currently in operation^18^. One region that may develop into a relevant site for CCS projects is the Paraná basin in southern Brazil, where voluminous layers of continental flood basalts^19,20^ cover an area of 1.2 × 10^6^ km^2^. The studied basalts represent the main tholeiitic magmatism record in the south portion of the South American platform^21–24^, partially covering central and southern Brazil, Uruguay, and Paraguay^19,20^ (Fig. 1A,B). These basalt lavas are grouped into the Serra Geral Group, which reaches a thickness of 1750 m in the central north portion of the basin^20,26,27^. They are comprised of heterogeneous lava packages with thin sedimentary interbeds^19,20^. The more primitive basaltic layers stratigraphically overlie aeolian sandstones, followed by andesitic, dacitic and rhyolitic lavas; the upper stratigraphic unit is formed by a basaltic flow emplaced during the waning phase of volcanic activity^24,25,28^.

Although there are previous studies on the potential for CO_2_ storage in basalts of the Paraná Basin^18,21^, no published experimental study has monitored the interaction between a dissolved CO_2_ solution and basalt rock, especially concerning the precipitation of carbonates. Such a laboratory experiment would provide details on the time required for carbon mineralization, the textural and mineral relationships, the estimated CO_2_ storage yields, and the influence of parameters such as pH, pressure, and the mineral assemblage of basalts.

## Methods

### Setup for the CO_2_ mineralization experiment

**Figure 2:**
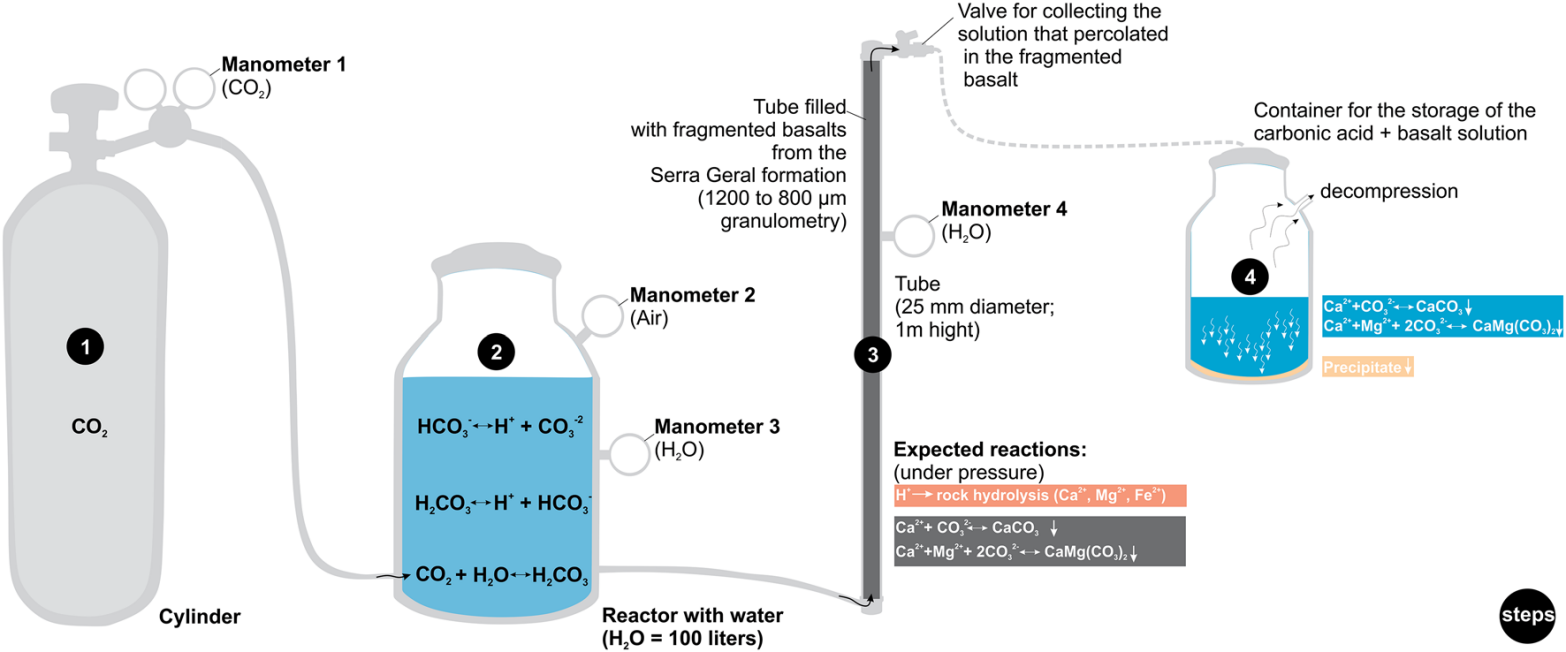
Schematic diagram of the experimental setup to test the reactive potential of South American basalts to CO_2_ storage. The steps are described in the “Setup for the CO_2_ Mineralization experiment” section.

Carbon mineralization occurs when CO_2_ is dissolved in water and reacts with Ca-rich minerals from basalts to form stable carbonate minerals^2,4,6,15^. Based on this premise, the material and setup requirements for the experimental mineralization test (Fig. 2) were designed based on data available in the literature and results of the Carbfix projects^2,3,5^ and Wallula project Columbia River CO_2_ injections^17^. In this context, to set up the CO_2_ mineralization experiment, we used a high-pressure CO_2_ tank (25 kg) to deliver a pressure of 1 bar (approximately 14.5 psi) of CO_2_ into a closed water container (100 L) (Fig. 2, steps 1 and 2). The reaction of CO_2_ with H_2_O formed carbonic acid and bicarbonate solution according to the following reaction (Eq. 1):

1 $$ {\text{CO}}_{{2}} + {\text{ H}}_{{2}} {\text{O}} \rightleftharpoons {\text{H}}_{{2}} {\text{CO}}_{{3}} \rightleftharpoons {\text{H}}^{ + } + {\text{ HCO}}_{{3}}^{ - } \rightleftharpoons {\text{2H}}^{ + } + {\text{ CO}}_{{3}}^{{{2} - }} . $$

Afterwards, the carbonic acid solution was transferred from the pressurized container into a polyvinyl chloride tube (Fig. 2, step 3) previously filled with basalt grains of 800–1200 µm diameter. The carbonic acid solution that percolated through the spaces between the basalt grains was collected daily for analysis in two different ways. The first method was conducted early in the morning after the basaltic grains had interacted with the carbonic acid solution under static conditions for almost 24 h ("closed system"). The second collection was performed after flushing the basalt grains for an hour with a new carbonic acid solution under a flow rate of 1 L/h, 0.8–0.9 bar, and at room temperature (25 °C). We named this second sampling procedure dynamic fluid-rock interaction as the “open system”. In both cases, the solution was collected using a valve placed at the end of the experimental tube (Fig. 2, step 3) and stored in a container (Fig. 2, step 4). This above procedure was repeated for 30 days, after which the tube filled with basalt grains was opened and checked for the presence of precipitated material. The supplementary materials provide detailed information on the analytical procedures used to characterize the basalt before and after interacting with carbonic acid solution.

### Sampling, field features and mineral assemblage of basalt before CO_2_ injection

**Figure 3:**
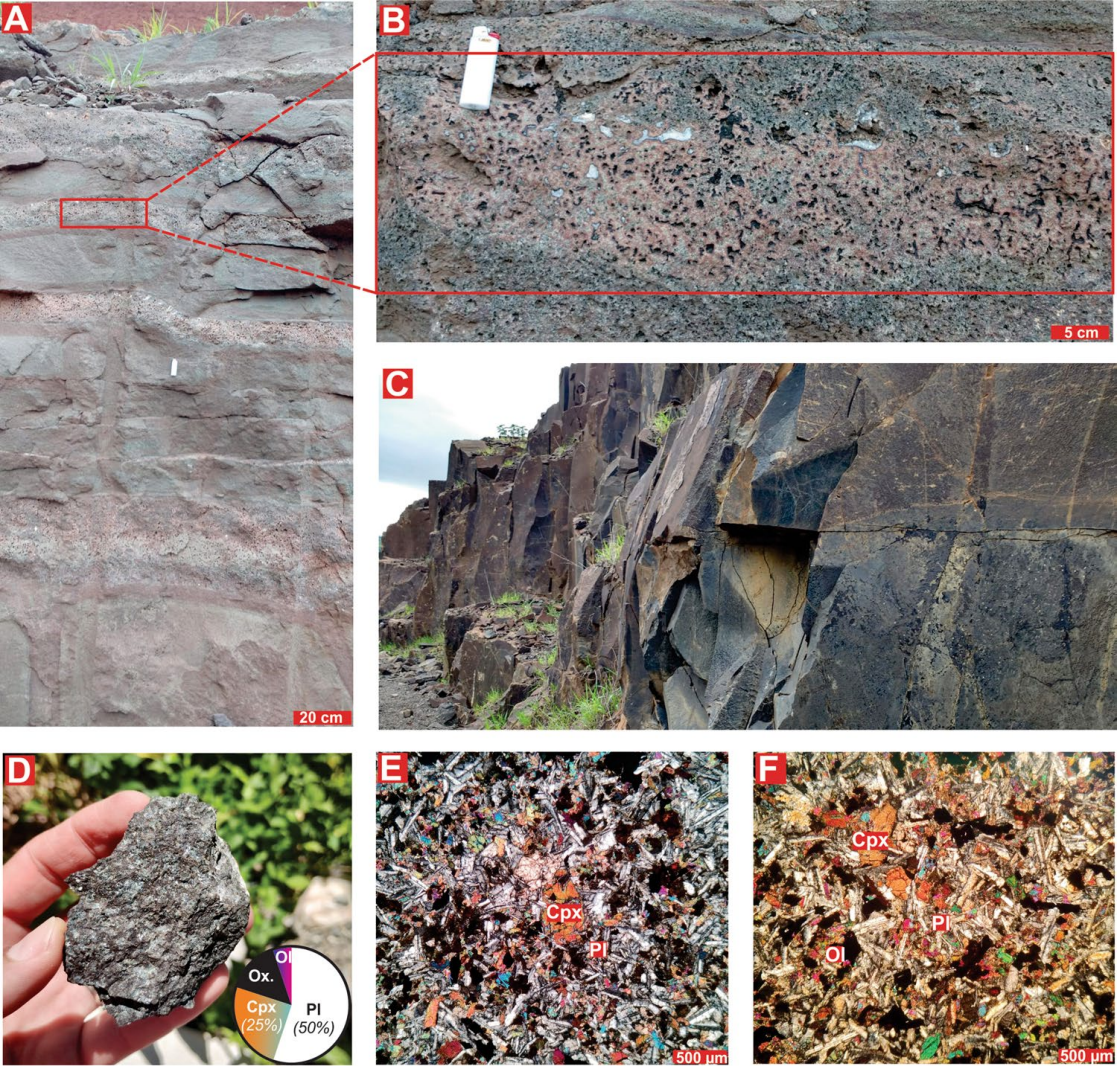
Field and petrographic features of studied basalts. (**A**–**C**) Basalt layers features in the Jataí region, central Brazil. (**D**–**F**) Detailed mineral textures and modal proportion of the basalt mineral assemblage.

Geological sampling in central Brazil was conducted to investigate and collect (see location in Fig. 1B) the representative rocks of the Paraná continental flood basalts. The basalts studied for the CO_2_ mineralization experiment ware collected at the northern limit of the Paraná continental flood basalts (Fig. 1A,B)^20,26–28^. The basalt outcrops display massive layers (Fig. 3A), usually interbedded with layers exhibiting high vesicular content (Fig. 3B). The massive basalt layers display pairs of NE and NW vertical and horizontal fractures (Fig. 3C). The mineral assemblage of the basalt layers includes plagioclase (45–55%), clinopyroxene (15–25%), olivine (2–5%) and Fe–Ti oxides (5–15%) as magnetite and ilmenite (Fig. 3D–F). The basalt samples used in the experiment do not have any veins or discrete carbonate crystals. Plagioclase with 12–16 wt.% CaO and clinopyroxene with 21–23 wt.% CaO (10–12 wt.% MgO) are the major calcium-bearing minerals (7.2–10.8 wt.% CaO in whole rock composition)^20,28^. Since these minerals represent 60–80% of the reactive surface of basalt (Fig. 3D), the studied crystalline basalts have a high potential for dissolution and release of Ca^2+^.

### Analysis of experimental results

#### Chemistry monitoring

**Figure 4:**
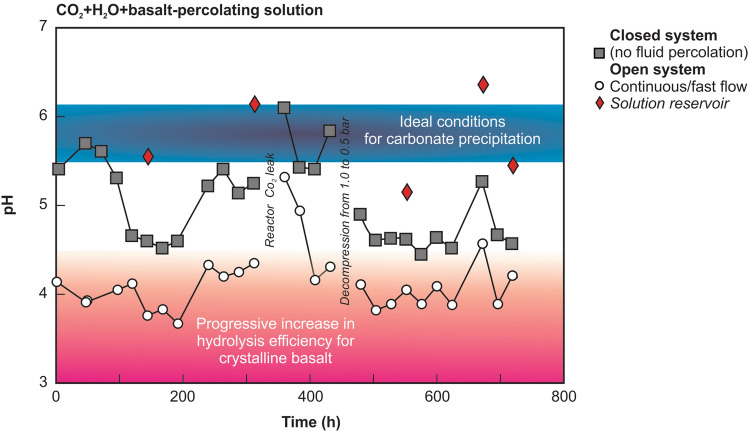
pH values of the carbonic acid solution that percolated into the basalt grains during the CO_2_ injection experiment. An open system represents a constant flow of 1 L/h during 1 h, whereas a closed system represents a stationary mode for 23 h (without constant flow/outflow in the experiment).

Chemistry and pH analyses of the carbonic acid solution that percolated into the basalt grains (Fig. 2, step 3) were carried out daily to monitor the dissolution efficiency throughout 30 days (720 h) (Supplementary Data 1). For each analysis, 20 mL samples were collected. The pH analyses indicate that optimal conditions for basalt leaching were achieved at pH = 3.89 (± 0.1)^3,29^ during the continuous flow regime (pH 3.5–4.5 in the open mode) (Fig. 4A,B). In contrast, under the closed system conditions, we observed higher pH values of 4.6–6.1 (Fig. 4A,B).

**Figure 5:**
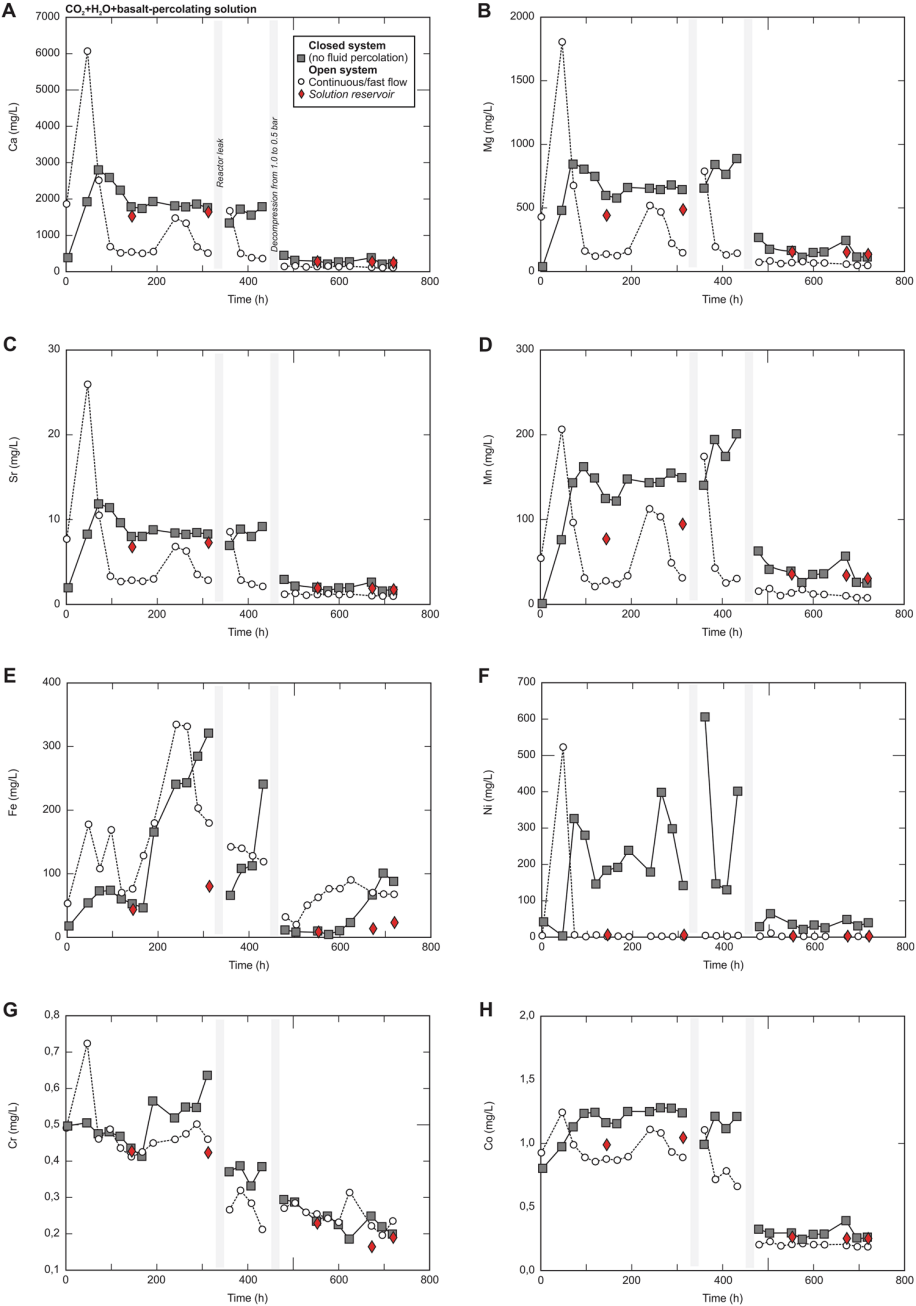
Chemistry composition of the carbonic acid solution that percolated into the basalt grains during the CO_2_ injection experiment. An open system represents a constant flow of 1 L/h during 1 h, whereas a closed system represents a stationary mode for 23 h (without constant flow/outflow in the experiment).

Our chemical analyses reveal the release and transport of Ca, Mg and Sr with a leaching peak at 48 h during the open system mode (Fig. 5A–C). After 48 h, the concentrations of these elements in the open system were reduced by around 2–3 times. In contrast, the concentrations in the closed system mode became systematically higher compared to the open system (Fig. 5A–H). It is important to highlight that releasing elements (leaching), such as Ca and Mg, limits the effective CO_2_ mineralization in basalt^4^. Our results also indicate that carbonic acid hydrolysis and leaching of basalt were effective and fast (48 h). Only Fe showed long times for leaching (after 288 h, Fig. 5E), possibly because Fe-rich minerals (i.e., oxides and olivine) have slower leaching ratios when compared to Ca- and Mg-rich minerals (i.e., plagioclase and clinopyroxene)^29^. Another hypothesis is that the higher Fe concentrations recorded during the later phases do not necessarily result from slow dissolution kinetics. Instead, the Fe-rich phases may have either become more accessible to reaction during the latter stages of the experiment or were equally reactive during earlier stages, but secondary mineralization incorporated the dissolved Fe effectively such that Fe concentrations were relatively low during the earlier stages. It is still possible that Ca- and Mg-rich minerals were dissolved to the same degree during the later stages. Still, their lower concentrations during these stages may result from more effective secondary mineralization at this time. Additionally, it is also possible that secondary mineralization that incorporated Ca and Mg was sluggish initially but improved significantly as more nucleation sites became more prevalent throughout the basalt. Indeed, different dissolution and precipitation processes can occur as the fluid percolates through the basalt grains^3,5,6^. Lastly, an important fact is that on the 18th day, the experiment lost pressure (decompression from 1.0 to 0.5 bar) and, as a direct result, the removal of elements from basalt by carbonic acid was reduced, indicating that the dissolution of basaltic reservoirs increases with pressure (Supplementary Data 1).

#### Precipitation formation and characterization

**Figure 6:**
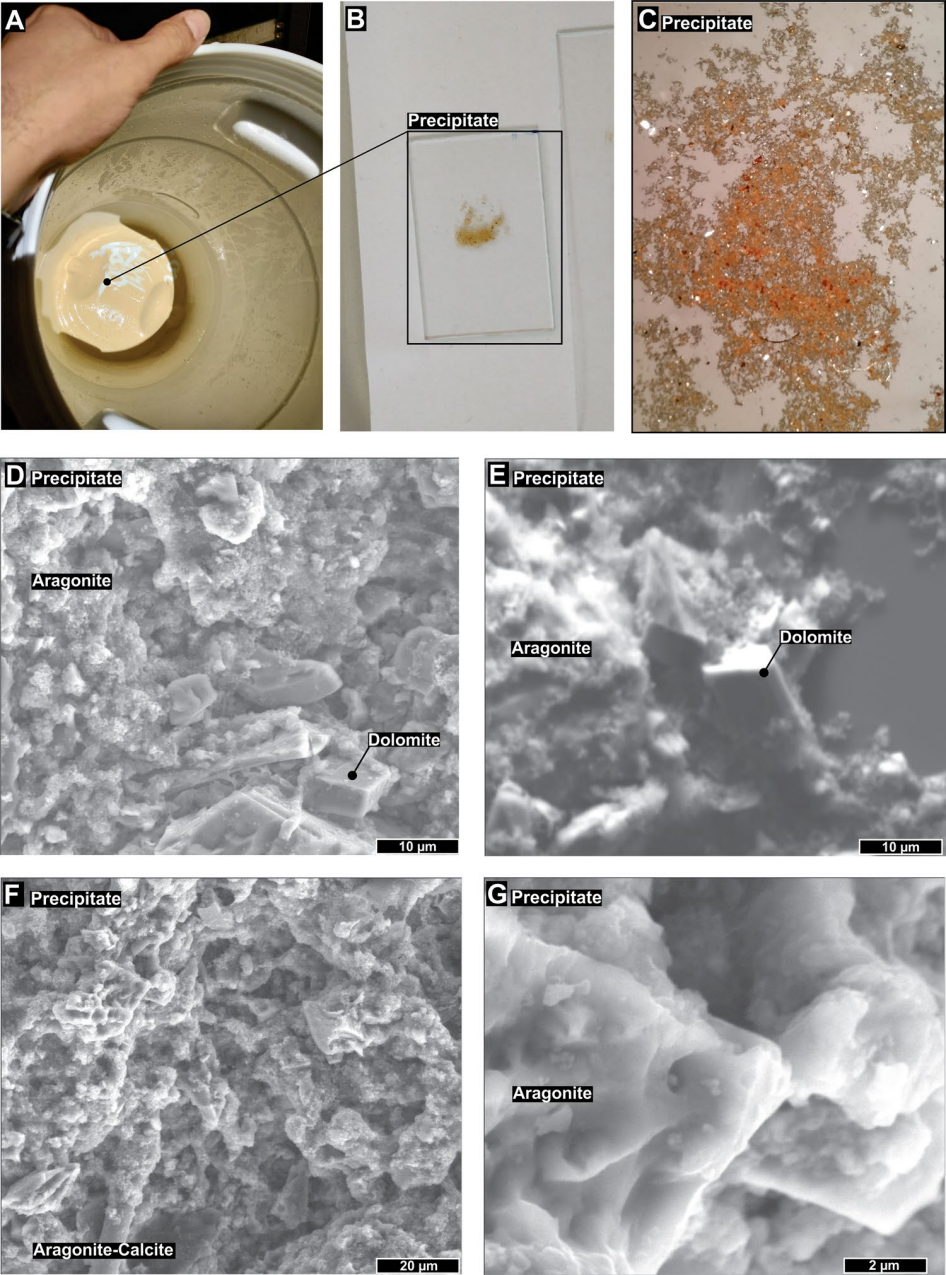
(**A**–**C**) Solution reservoir (Fig. 2, step 4) with a thin layer of precipitate after 18 days. (**D**–**G**) Backscattered electron images showing that the precipitate consists mainly of trigonal and orthorhombic crystals surrounded by a matrix of cryptocrystalline crystals.

**Figure 7:**
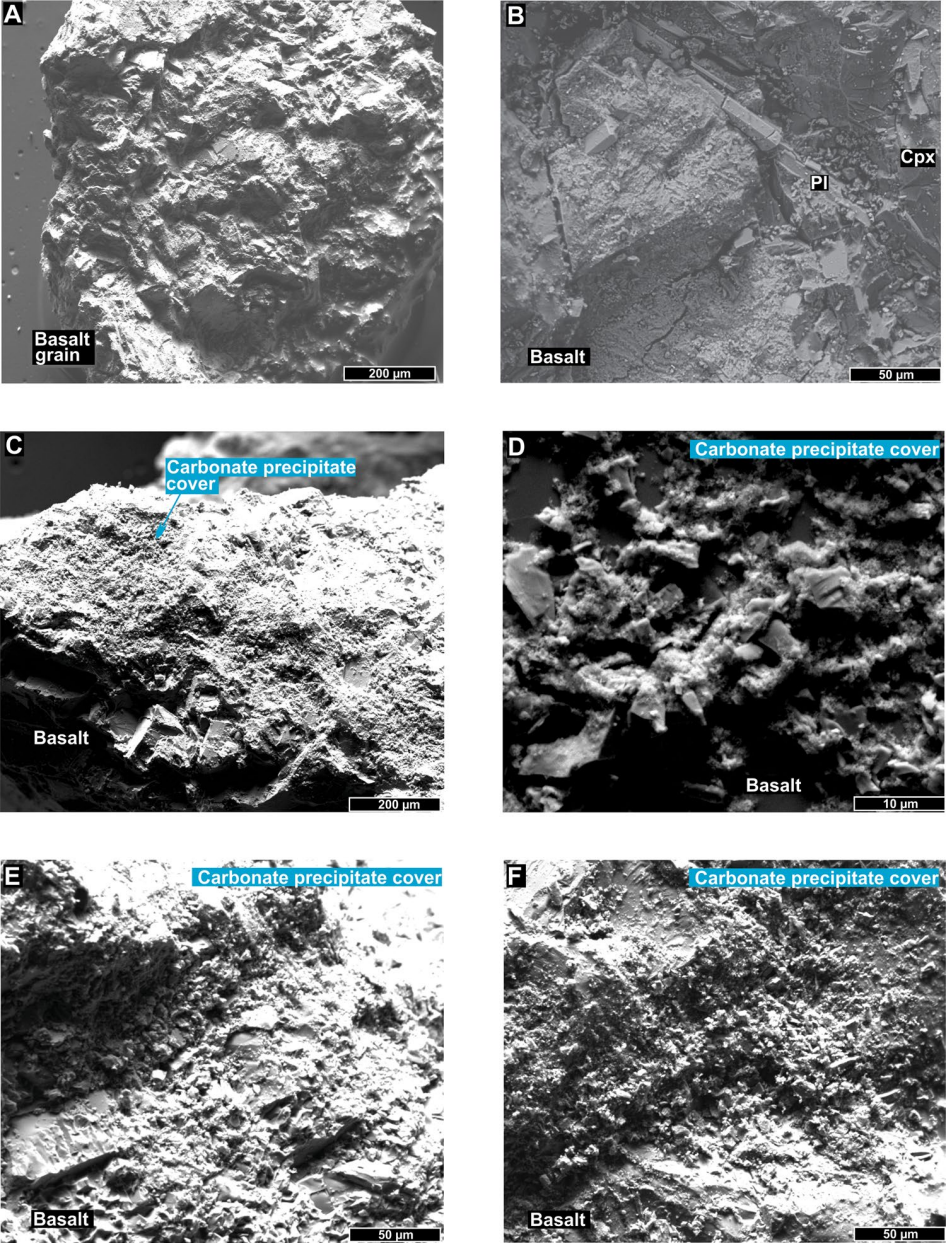
(**A**–**F**) Backscattered electron images showing precipitates formed by crystals similar to those observed in the solution reservoir (Fig. 6) covering the basalt grains (Fig. 2, step 3) after 30 days.

After 72 h (3 days) of the experiment, the carbonic acid solution that percolated through the basalt began to present a thin layer of precipitate at the bottom of the reservoir (Fig. 2, step 4). After 432 h (18 days), a well-defined layer of light brown to beige precipitate was formed (Fig. 6A,B ). Indeed, the pH of this solution increased from 5.15 to 6.36 in the solution reservoir (Fig. 4B), favoring carbonate precipitation^2,5,29,30^. Furthermore, texture and crystallographic analysis in an optical microscope (Fig. 6C) and scanning electron microscopy (SEM) (Fig. 6D–G) revealed that the precipitate consisted mainly of trigonal calcite crystals with very high birefringence surrounded by a matrix of cryptocrystalline aragonite crystals (Fig. 6D–G). Most crystals were twinned growths of individual crystals that formed pseuohexagonal trilling (Fig. 6F,G). Most precipitated crystals were generally hexagonally shaped due to the twinning like that described for the aragonite-calcite precipitation mechanisms^31^. After 720 h (30 days) of continuous experiment, the tube filled with basalt grains (Fig. 2, step 3) was opened, and the same precipitate textures were observed on the surface of the basalt grains (Fig. 7A–F). This result was expected since during the closed mode (without continuous flow percolation), the pH usually reached values above 5.5, which allowed carbonate precipitation.

#### Fourier transform infrared spectrophotometer (FTIR) and X-ray diffraction (XRD)

**Figure 8:**
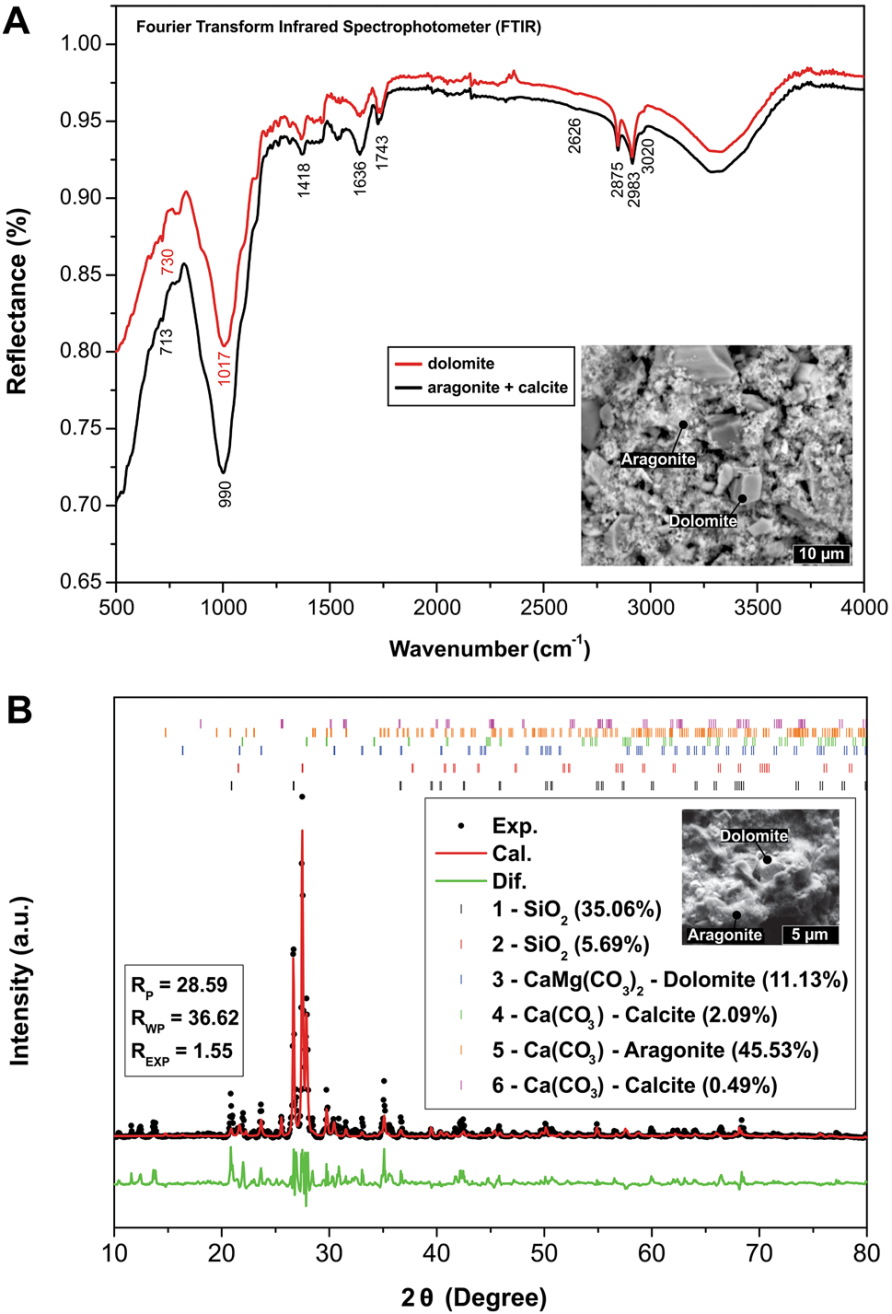
(**A**) FTIR spectra of precipitate formed by aragonite and dolomite. The absorption features at 3020, 2626 and 730 cm^−1^ are characteristic of dolomite, whereas the other absorption values are characteristics of combined calcite-aragonite and dolomite^33,34^. The strong absorption bands observed at 1017–990 cm^−1^ may be associated with glass-fiber wool fragments in the precipitate (*fragments of glass wool used to immobilize the basalt grains were found with the mineral precipitate). (**B**) X-ray diffractogram of the CO_2_ precipitate. The black balls represent the experimental points, the solid red line represents the refinement obtained using the Rietveld method, and the solid green line represents the difference between the experimental points and the fit. The bars represent the Bragg angles for the phases found. R_P_, R_WP_ and R_EXP_ represent the goodness of fit.

The FTIR patterns obtained of the precipitated material in the solution reservoir show two well-defined signatures with strong absorption bands between 3020 and 2875, 2626, 1743, 1636, 1418, 730 and 713 cm^−1^ (Fig. 8A) (Supplementary Data 2). These patterns are compatible with the presence of aragonite + calcite and dolomite^32,33^. Compared to aragonite + calcite, dolomite displays characteristic FTIR absorptions at 3020 cm^−1^, 2626 cm^−1^ and 730 cm^−1^; the presence of these absorption bands helps indicate the presence of dolomite^34^ (Fig. 8A). Specifically, the band at 730 cm^−1^ is related to the in-plane bending mode of CO_3_^2−^^33,34^.

The X-ray diffraction analyses revealed the presence of at least 6 crystalline phases in the experiment precipitate (Fig. 8B) (Supplementary Data 3 and 4): 36.02% and 6.19% of SiO_2_ (two quartz phases), 11.18% of CaMg(CO_3_)_2_ (dolomite), 43.95% of Ca(CO_3_) (aragonite), 2.15% and 0.51% of Ca(CO_3_) (two calcite structures). The quartz (SiO_2_, 42.21%) is related to the glass wool used during the experiment to retain the basaltic grains in the tube (Fig. 2, step 3). FTIR and XRD results are consistent with the aragonite-calcite and dolomite patterns (Fig. 8A,B)^35^. Therefore, the CO_2_ capture experiment yielded a precipitate comprising 75.95% aragonite, 19.34% dolomite, and 4.60% calcite mineral assemblage.

## Discussion

### Basalt dissolution and CO_2_ carbonation

Laboratory and field studies have shown that basalt formations could be secure repositories for anthropogenic CO_2_ emissions^1,4,6^. Specifically, the CarbFix project mineralized over 60% of the injected CO_2_ within four months of injection^5^. Furthermore, increased greenhouse gas injection rates also accelerated the rates of CO_2_ mineralization^4,5,11^. In basaltic reservoirs, mineral dissolution rates increase dramatically under low pH conditions near the CO_2_ injection point of mineral carbonation experiments^1,2,4,5^. In general, dissolution rates in aluminium-rich minerals and rocks (i.e., labradorite and basalts) are slower at neutral pH and increase again at higher pH^3,5,6,36^. On the other hand, the rates of aluminium-free minerals, such as olivine (forsterite) and pyroxene (diopside), continuously decrease with increasing pH, so the dissolution of these minerals is slow under conditions in which carbonates tend to precipitate^3,5^.

Our results also indicate that crystalline basalts have high reactivity due to primary magmatic minerals such as Ca-rich plagioclase and clinopyroxene being more easily dissolved by carbonic acid than supergene minerals^4–6,11^. This implies that supergene and alteration minerals reacted with hydrothermal or weathering fluids, consequently losing the capacity for the maximum release of cations. Basalt's natural porosity, combined with physical stimulation related to CO_2_ injection, increases the basalt dissolution rates due to increased surface contact between the carbonic acid and host rock^10,11^. According to the literature, temperature plays a significant role. For instance, an increase from 0 to 100 °C implies a CO_2_ dissolution ratio increase from 4.5 to 60 times^1,4,36^. Regarding porosity, the basaltic lava layers have an average porosity of 8%, with higher values at the base and top layers (up to 45%) and lower values at the central portions of the layers (5%)^6,20,37^.

The general decrease in element contents under a closed system in the first days of the experiment (i.e., without constant fluid percolation) indicates that dissolution of basaltic minerals tends to decrease with increasing pH from 3.6 to 6.2^3^. This suggests that reduction of the carbonic acid flow promotes a balance between the injected solution and host basalt, raising the pH. Since high pH values (5.5 and 6.5) are ideal for carbonate precipitation^1,2,37^, this process occurs away from the CO_2_ injection site and under lower fluid pressures^3^.

Petrographic and XRD analyses indicate that 20% of the precipitate is made of large Mg-calcite crystals (10–30 µm) immersed in a mass made of 80% cryptocrystalline aragonite (< 5 µm) (Figs. 6, 7, 8). These analyses also suggest that aragonite and calcite have quite distinct precipitation kinetics, i.e., the growth rate of aragonite was much higher than that of calcite, as previously reported by other studies^31,38^. The higher proportion (~ 80%) of cryptocrystalline aragonite crystals (< 5 µm) compared to larger trigonal-rhombohedral dolomite crystals (~ 20%) (10–30 µm) possibly occurs due to aragonite precipitating faster at 25 °C^31,38^ (Fig. 6D–G). In this reaction, it is also frequent to incorporate divalent cations (i.e., Sr^2+^) into aragonite^38^. The incorporation of Mg occurs preferentially in overgrowth crystals of calcite (magnesium calcite) independent of the precipitation rate^38^. Incorporation of Mg into calcite increases in low-saline (low NaCl content) solutions such as those used during the experiment, which may explain the high concentrations of Ca-dolomite to high Mg-calcite transition^38^. Indeed, dolomite is more stable than Mg-calcite as pH declines^39^. Lastly, as mineral composition reflects the susceptibility to basalt dissolution, clinopyroxene leaching can increase the availability of magnesium ions for carbonate precipitation^5,6^.

### CO_2_ storage yield

The solubility constant (K_sp_) of aragonite (6.0 × 10^–9^) is higher compared to its polymorph, calcite (3.31 × 10^–9^)^40^. Therefore, as the molar concentration of Ca during the experiment oscillated from 0.109 to 6.012 mg/L (Supplementary Data 1), a higher proportion of aragonite (75.4%) compared to calcite (4.6%) in the precipitate was not expected (Fig. 8A). However, the coexisting presence of Mg in solution reduces the reaction kinetics of calcite precipitation^41,42^, which explains the higher volume of aragonite in the precipitate. According to the expected reactions (Fig. 2, step 3), precipitation of aragonite or calcite requires 1 mol of calcium to 2 mol of bicarbonate. Since the aqua-carbonic solution interacted with the basalt, the limiting factors for carbonate precipitation were pH and Ca^2+^ ions in the solution (minimum Ca values to initiate precipitation were reached 48 h after carbonic acid injection; Fig. 5A). Likewise, with the pH around 3.89 at 1 bar, the mean concentration of CO_2_ is 1.22 mg/L; therefore, considering this solubility of CO_2_, the Ca content (removed from basalt) required to initiate precipitation is around 6500 mg/L. Any Ca content near this value tends to initiate carbonate precipitation. Therefore, the higher Ca content in the system did not exceed 6.069 ppm even with the continued input of calcium into the reservoir (Fig. 5A). Calcium availability is a key (limiting factor) issue regarding CO_2_ mineralization in basalts^4^, given the continuous injection of CO_2_. In our experiment, the difference between Ca content under high pressure conditions (6069 mg/L) and low-pressure conditions (1527 mg/L), may be used to estimate the amount of precipitated carbonate. For estimation of the yield, we have considered the following assumptions: (i) Ca^2+^ ions were the limiting component in the precipitation process; (ii) precipitation of carbonate occurred only after the reaction of Ca^2+^ and HCO_3_^-^ ions (approach for chemical estimation purposes); and (iii) yield was estimated based on the difference of Ca^2+^ ion per unit volume after extraction (initially at 6069 mg/L) and carbonate precipitation (depletion to 1527 mg/L). It implies that at least 74.8% of dissolved Ca reacted with HCO_3_^−^ to precipitate carbonate 48 h after starting the experiment.

Regarding dolomite, although it presents a lower K_sp_ (mean of 8.12 × 10^–18^), this mineral constitutes a volume of 19.6% in the CO_2_ precipitate, reflecting its more complex kinetics (known as the “dolomite problem”)^42^ combined to Mg availability (2.43 × 10^–3^ to 1.803 mg/L; Supplementary Data 1) during the experiment for its formation (Ca^2+^  + Mg^2+^  + 2CO_3_^2−^ ⇌ CaMg(CO_3_)_2_). Uncertainty in the determination of K_sp_ and the scarcity of present-day dolomite formation is to be expected because of the known kinetic inhibition of precipitation of dolomite at low temperatures^41,42^.

### CO_2_ storage potential in South America

The dissolution of the CO_2_ in water before or during injection results in immediate solubility trapping^1,4,5,43^. Since the injected gas-charged fluid is denser than the CO_2_-free water, it tends to sink rather than rise to the surface, reducing the risk of leaks^2,13,37,43^. Although CO_2_ dissolution requires a significant amount of water (Eq. 1), the method is simple and cost-effective^2,4,7^. Carbonic acid is a natural solution formed elsewhere on Earth by reacting water and dissolved CO_2_. Indeed, carbonic acid is found on rainwater and plays a major role in weathering processes and the mobility of metals on the Earth’s surface^43,44^. Furthermore, throughout the history of the Earth, approximately 99.9% of CO_2_ has been removed from the atmosphere through the weathering of rocks^44,45^. The bicarbonate solutions resulting from this weathering are transported by rivers to the sea, where marine organisms convert them into carbonate rocks^44–46^. Likewise, the higher the pressure conditions, the lower the mass of water required and the higher the CO_2_ dissolution rates, which accelerates the mineral carbonation process^3–5,10,13^.

Geological factors such as reactivity, interconnectivity, fracture systems, pre-existing fluid (water or hydrocarbon), and structural and stratigraphic traps are determinants for CO_2_ storage in basaltic reservoirs^1–3,6,10^. Thus, the first experimental results show that Paraná continental flood basalts have mineralogical, chemical, and petrophysical properties that are efficient for rapid and definitive CO_2_ geostorage. Indeed, the CarbFix results indicate that 72 ± 5% of the injected CO_2_ was mineralized to carbonate minerals^8^. Lastly, according to our experiment, Ca-rich mineral assemblage (~ 75% of the basalt), formed mainly by plagioclase and Ca-rich clinopyroxene, releases Ca^2+^ under pH values around 3.89, 1.0–0.5 bar, and at 25 °C. We further show that carbonate precipitation occurs at pH values between 5.52 and 6.14 under low CO_2_ pressure conditions.

## Conclusion

The carbonic acid reacted with basalt and formed stable carbonate minerals such as aragonite-calcite (CaCO_3_) (80%) and dolomite (MgCa(CO_3_)_2_) (20%). Thus, the proposed experiment efficiently converted CO_2_ from a gas phase into a crystalline solid permanently stored in the subsurface. Furthermore, our findings demonstrate that converting CO_2_ into carbonate minerals within basalt rocks takes only a few days. Likewise, this is the first experiment demonstrating the safe long-term storage potential of anthropogenic CO_2_ emissions through precipitation of carbonates in the Paraná continental flood basalts of South America.

### Supplementary Information

Supplementary Information 1.

Supplementary Information 2.

Supplementary Information 3.

Supplementary Information 4.

Supplementary Information 5.
