# Supplementary material for: Unraveling the rapid CO2 mineralization experiment using the Paraná flood basalts of South America
Source: Sci Rep. 2024 Apr 6;14:8116. doi: 10.1038/s41598-024-58729-w (PMC10998914; doi:10.1038/s41598-024-58729-w)
Supplement: Supplementary file 4 — Supplementary Information 4. [file 41598_2024_58729_MOESM4_ESM.docx]

Supplementary Data 04. Atomic positions, lattice, and crystallographic parameters were used for XRD data.

﻿Atomic positions, lattice parameters, density and volume of the compound Phase 3 – CaMg(CO_3_)_2_ (dolomite) with trigonal structure and space group R-3 number 148. The values in parentheses represent the estimated error in the last digits.

|  | Phase 3 – CaMg(CO_3_)_2_ | | | |
| --- | --- | --- | --- | --- |
| Atom | Ca | Mg | C | O |
| Site | 3a | 3b | 6c | 18f |
| *x* | 0 | 0 | 0 | 0.0110(369) |
| *y* | 0 | 0 | 0 | 0.4973(1996) |
| *z* | 0 | 1/2 | 0.2236(163) | 0.1774(170) |
| *a=b (Å)* | 4.9016(4) | | | |
| *c (Å)* | 16.2695(23) | | | |
| *d (g/cm^3^)* | 1.593 | | | |
| *V (Å^3^)* | 338.524(62) | | | |

Atomic positions, lattice parameters, density and volume of phases 4 and 6 – Ca(CO_3_) compounds (calcite) with trigonal structure and space group R-3c number 167. The values in parentheses represent the estimated error in the last digits.

|  | Phase 4 – Ca(CO_3_) | | | Phase 6 – Ca(CO_3_) | | |
| --- | --- | --- | --- | --- | --- | --- |
| Atom | Ca | C | O | Ca | C | O |
| Site | 6b | 6a | 18e | 6b | 6a | 18e |
| *x* | 0 | 0 | 0.2208(742) | 0 | 0 | 0.2138(1792) |
| *y* | 0 | 0 | 0 | 0 | 0 | 0 |
| *z* | 0 | 1/4 | 1/4 | 0 | 1/4 | 1/4 |
| *a=b (Å)* | 5.2525(15) | | | 6.9494(33) | | |
| *c (Å)* | 18.0350(53) | | | 17.1293(47) | | |
| *d (g/cm^3^)* | 2.222 | | | 1.336 | | |
| *V (Å^3^)* | 430.910(218) | | | 716.418(513) | | |

﻿

Atomic positions, lattice parameters, density and volume of the Phase 5 – Ca(CO_3_) compound (aragonite) with orthorhombic structure and Pnma space group number 62. The values in parentheses represent the estimated error in the last digits.

|  | Phase 5 – Ca(CO_3_) | | | |
| --- | --- | --- | --- | --- |
| Atom | Ca | C | O(1) | O(2) |
| Site | 4c | 4c | 4c | 8d |
| *x* | 1/4 | 1/4 | 1/4 | 0.5765(374) |
| *y* | 0.4389(40) | 0.7905(3710) | 0.8203(757) | 0.6955(218) |
| *z* | 0.7721(38) | -0.0658(1311) | -0.0638(508) | -0.0436 (115) |
| *a (Å)* | 5.0607(13) | | | |
| *b (Å)* | 9.1003(14) | | | |
| *c (Å)* | 7.9948(6) | | | |
| *d (g/cm^3^)* | 0.638 | | | |
| *V (Å^3^)* | 368.191(112) | | | |
