# Supplementary material for: Unraveling the rapid CO2 mineralization experiment using the Paraná flood basalts of South America
Source: Sci Rep. 2024 Apr 6;14:8116. doi: 10.1038/s41598-024-58729-w (PMC10998914; doi:10.1038/s41598-024-58729-w)
Supplement: Supplementary file 5 — Supplementary Information 5. [file 41598_2024_58729_MOESM5_ESM.docx]

# Supplementary methods and analytical procedures

## Basalt sampling and petrography

Geological sampling in central Brazil was conducted to investigate and collect (see location in Fig. 1B) the representative rocks of the Paraná continental flood basalts. Samples from basalt layers were cut in systematic thin sections relative to the foliation and investigated in the Microscopy Laboratory of the Institute of Geosciences from University of Brasília (Brazil). Most representative basalts in thickness were selected for detailed petrographic descriptions (Fig. 3A-F).

## Electron microscopy imaging

Petrographic investigation of the basalts and CO_2_ precipitate was based on optical microscope and Cathodoluminescence (CL) and backscattered electron (BSE) images collected using a Zeiss Merlin FE-SEM of the Institute of Geosciences of the University of Brasília (Brazil). Conditions during imaging were 10 kV accelerating voltage, 11 nA probe current, with a working distance of 9.5 mm. Major and trace element analyses of minerals were conducted using a Zeiss EVO SEM equipped with an Oxford Instruments × Max 20 mm2 EDS detector, an Oxford Instruments Wave Dispersive X-ray Spectrometer and Oxford INCA software. Beam conditions during quantitative analysis were 20 kV accelerating voltage, 1.0 A probe current, with a working distance of 8.5 mm and a specimen beam current of 20.00 nA. The counting time was 10 s live time for the EDS detector and 60 s on peak, with 30 s off-peak for the WDS detector.

## Chemistry analysis

Major and trace element analyses of the carbonic acid solution that percolated into the basalt were determined (Supplementary Data 01) by inductively coupled plasma mass spectrometry (ICP-MS, 7500 Series, Agilent). The instrument was operated with a nebulization system composed of a pneumatic microconcentric nebulizer and a cyclonic chamber, with a nebulizer gas flow of 1 L/min. Before the element determinations an instrumental check was carried out (Tuning) using a solution containing 10 µg/L of Li, Y, Tl and Ba. The isotopes monitored during the analysis were, Ca^43^, Mg^24^, Ba^137^, Fe^57^, Ni^60^, Co^59^, Mn^55^, Cu^63^, K^39^, Sr^88^ and Cr^53^; furthermore, the isotope In^115^ was used as internal standard, wherein a solution of In was added in all solutions at concentration of 10 µg/L. For the instrumental calibration, suitable dilutions of a multielemental standard solution containing all the analytes in the concentration of 1000 mg/L (SpecSol, São Paulo) were used. All the ICP-MS analysis were carried out in triplicate and from the results were subtracted the blank values.

## Fourier Transform Infrared Spectrophotometer

Fourier Transform Infrared Spectroscopic analysis (FTIR) is a potential method for acquiring qualitative and quantitative information about sample mineralogy^48^. Spectroscopy can be used to identify materials that are amorphous or poorly crystalline^47,48^. FTIR spectra of the CO_2_ precipitate generated during the experiment are shown in Supplementary Data 02. The equipment used was FTIR (4000-400 cm^-1^) equipped with a TA DLaTGS detector of the Physics Institute of the University of Brasília. FTIR is based on changes in vibrational energetic states caused by photon-matter interaction in the infrared region (100 to 10000 cm^-1^)^48^.

## X-Ray diffraction

The precipitate material was examined by X-ray Diffractometer, Malvern Panalytical Empyrean (XRD) at the X-Ray Diffraction Laboratory of the Physics Institute of the University of Brasília. The XRD allows materials to be characterized by different atomic arrangements in the crystal lattice. Reading occurs along the diffracted line for each set of crystal planes. The graph generated relates the intensity of the diffracted lines as a function of the diffraction angle. This system uses X-ray tubes with Cu-Kα radiation with wavelengths 1.5405980 *Å* and 1.5444260 *Å* for lines *α_1_* and *α_2_*, respectively. The measurement was performed in the range of 10 to 80° with a step of 0.05°. The refinement using the Rietveld method was done considering function 1 (Lorentzian) of the DBWS9411 program given by:

$\frac{\sqrt{C_{1}}}{{2H}_{k}}\left( 1+\frac{C_{1}\left( 2\theta_{i}-2\theta_{k} \right)^{2}}{{H_{k}}^{2}} \right)$,

where *C_1_* is a constant, *H_k_^2^* is the half-height width of the diffraction peak for the k-th reflection, and *2θ_i_ - 2θ_k_* is the Bragg angle for the kth reflection.

The result of the X-ray diffraction analysis was compared with the crystallographic parameters of the minerals using the PowderCell program to assist in the initial identification of the mineral structure formed. In addition, the Match program, a crystallographic database, was also used to find possible formations of additional phases. All crystallographic parameters of the mineral phases used in X-ray diffraction analysis are shown in the Supplementary Data 3 and 4.
